# Interventions for Increasing Acceptance of New Foods Among Children and Adults with Developmental Disorders: A Systematic Review

**DOI:** 10.1007/s10803-019-04075-0

**Published:** 2019-05-23

**Authors:** L. R. Chawner, P. Blundell-Birtill, M. M. Hetherington

**Affiliations:** School of Psychology, University of Leeds, Leeds, LS2 9JT UK

**Keywords:** Selective eating, Eating behaviour, Dietary variety, Developmental disorder

## Abstract

People with developmental disorders (DD) often display high levels of selective eating, which can result in micronutrient deficiencies. It is therefore essential to explore ways to increase dietary variety in this population. To identify different types of interventions promoting increased acceptance of new foods or dietary variety for DD populations and to determine their effectiveness. Thirty-six studies met criteria for inclusion in the review. Twenty-two types of intervention were identified with 34 studies being reported as effective and 33 of these incorporating components drawn from learning theory. Multi-component interventions centred on operant conditioning, systematic desensitisation and changes to environment and familial practices were reported as effective for individuals.

Food selectiveness, or picky/fussy eating, can be defined as a lack of variety in the diet (Carruth et al. 1998) or as consumption of a limited number of foods (Rydell et al. 1995). In developmentally disordered (DD) populations, such as those with autistic spectrum disorder (ASD) or intellectual disability (ID), rates of food selectiveness (Williams and Seiverling 2010), eating related problem behaviours (Ledford and Gast 2006) and rejection of both novel and already accepted foods (Seiverling et al. 2018) are common. Consequently, food selectivity and refusal often results in people missing, or having very low levels, of important nutrients in the diet (Esteban-Figuerola et al. 2018; Sharp et al. 2013). This may lead to micronutrient deficiencies and in turn specific health consequences, such as reduced bone growth due to low calcium intake (Hediger et al. 2008) or scurvy due to low levels of vitamin C (Ma et al. 2016). However, these effects can be prevented through habitual intake of a varied diet and specifically by increasing intake of nutrient-dense fruit and vegetables (FV). Yet, feeding interventions with people with ASD often aim to increase the volume of food consumed and rarely consider increasing the variety of foods consumed (Marshall et al. 2015b).

Ledford et al. (2018) reviewed the various interventions used to treat feeding related behaviours in the ASD population. These methods included escape extinction (EE), fading techniques and positive reinforcement, among other strategies. The techniques are predominantly used in clinical or feeding disordered populations where fussy eating has caused other problems, such as malnutrition, growth faltering or failure to thrive (Barnhill et al. 2017). Fewer studies have assessed techniques used to promote more general components of healthy eating in DD populations such as increasing variety which have far less urgent clinical need than those used to address more serious protein-energy malnutrition or micronutrient deficiency but are nonetheless valuable to ensure optimal food intake.

In typically-developing populations, rejected foods often include nutrient-dense FV that may be bitter in taste or unusual in appearance (Dovey et al. 2008). Interventions designed to increase intake of FV in typically developing populations have been based on mere exposure effects (Barends et al. 2019; Nekitsing et al. 2018). However, repeated exposures are rarely used in the same format to increase healthy eating and variety in DD populations (e.g. storybooks or classroom games; Coulthard and Ahmed 2017; Heath et al. 2014). The current review therefore aims to identify interventions used with DD populations and to assess their effectiveness in promoting healthy eating behaviours including increasing dietary variety. This review focused on measures of increased intake of novel or previously rejected foods, increased number of foods consumed, food choice and whether interventions used have lasting effects. Secondary aims were to identify the settings in which these interventions were carried out, who the interventions were implemented by, what diagnoses the participants of interventions had and the types of study design used.

## Methods

### Registration

The protocol for carrying out this review was specified and registered in advance with the International prospective register of systematic reviews PROSPERO, registration number: CRD42019116769.

### Eligibility Criteria

The PICOS (participant, intervention, comparison, outcomes and study-design) framework was used to develop inclusion and exclusion criteria for the review. Studies were considered for inclusion if their sample consisted of participants of any age with a diagnosis of a DD. DD was used as a broad term to include cognitive or learning DD including ASD, ID, attention-deficit hyperactivity disorder (ADHD), or other disorders of a similar nature (e.g. developmental delay, Down’s syndrome). Studies were excluded from the review if they included participants with eating disorders (avoidant-restrictive food intake disorder was also excluded), failure to thrive (or tube-dependent participants), Alzheimer’s disease, dementia, diabetes or pregnancy. Participants with other mental health conditions or unrelated medical conditions were also excluded.

Included studies were those which had employed an intervention exposing participants to food stimuli, techniques to increase food intake or environmental changes around mealtime eating. Control groups for interventions were not restricted and studies were considered for inclusion if they used pre-intervention measures of food intake as a control for post-intervention measures.

Primary outcome measures related to healthy eating were acceptable. These included: amount of food eaten (weighed) if a variety of foods were introduced, number of different foods eaten, number or percentage of bites eaten and self-reported intakes. Studies were excluded if they did not report food intake, or if the amount or types of foods used in the intervention were not reported. Any study design was considered for inclusion dependent on whether an intervention was carried out.

Studies were also excluded if (1) only problem behaviours at mealtimes (e.g. tantrums, aggressive behaviours) were reported, (2) the full text was not in English or (3) the study was published before the year 2000. It was deemed that papers before this time may not be relevant to the current review as diagnostic criteria for DD were revised in this year (American Psychiatric Association 2000).

### Information Sources and Search Strategy

Published articles were identified through searching electronic databases and scanning reference lists of previous, similar reviews. Limits were placed on language (English) and year of publication (01/01/2000-07/11/2018). This search was applied to five databases, Ovid (MEDLINE 1996–present; EMBASE 1996–present and PsychInfo 2002–present), EBSCO (CINAHL 1960–present) and Web of Science (core collection 1900–present). All searches were conducted on 07/11/2018. A full list of search terms based on the PICOS criteria are provided in Table 1 and were used to search all databases. Search terms for each relevant PICOS criterion were adapted from search terms used by previous systematic reviews (Brown et al. 2016; Brylewski and Duggan 1999; Sharp et al. 2017; Veltman et al. 2005; Williams et al. 2006).

**Table 1:** Search strategy of OVID (Medline, Embase and PsychInfo), EBSCO (CINAHL) and Web of Science (Core collection) based on PICOS criteria

1	ASD OR special need* OR autis* OR Asperger OR Autistic-Disorder OR Asperger-Syndrome OR developmental disability OR intellectual disability OR ID OR autism spectrum disorder* OR mental* OR handi* OR retard* OR learning disab* OR cognitive impair* OR developmental delay OR DD OR global dev* OR GDD OR pervasive develop* OR PDD OR ADHD OR attention deficit hyperactivity disorder OR attention deficit disorder OR ADD
2	Fussy eat* OR Picky eat* OR food neophobia OR food fuss* OR selective eat* OR food select* OR eating habit* OR Food phobia OR Food refusal OR ARFID OR avoidant-restrictive food intake disorder OR Avoidant restrictive food intake disorder OR feeding disorder OR Pediatric feeding disorder OR feeding problem OR feeding difficult* OR Paediatric feeding disorder OR unhealthy diet OR diet quality OR inappropriate mealtime behav* OR problematic mealtime behav* OR feeding difficult* OR mealtime or tantrum* OR faddy eat* OR food fad* OR food sensitive* OR food defensive OR food aversion OR eating problem OR food restrictive OR food type OR CEBQ OR CFQ OR SFQ OR EBQ OR ORI-CEBI OR CPEBQ
3	Healthy eating OR vegetable OR novel food OR experiential learning OR sensory learning OR experience OR applied behaviour analysis OR exposure OR ABA OR applied behavior analysis OR Behavioral intervention OR behavioural intervention OR Behavioral treatment OR behavioural treatment OR Intervention OR parent training OR nonremoval OR non-removal OR reinforcement OR reward OR punish* OR systematic desensitisation OR systematic desensitization OR SD OR escape extinction OR representation OR shaping OR fading OR teach* OR learn*
4	Willingness to try OR report OR recall OR food diar* OR weight OR weigh OR amount eaten OR food choice OR novel food OR food refus* OR eating behaviour OR eating behaviour OR diet OR health OR sensory sensitive* OR sensitivity OR defensive OR compliance OR eating OR bite* OR number of bite*
5	1 AND 2 AND 3 AND 4

### Study Selection

**Fig. 1:**
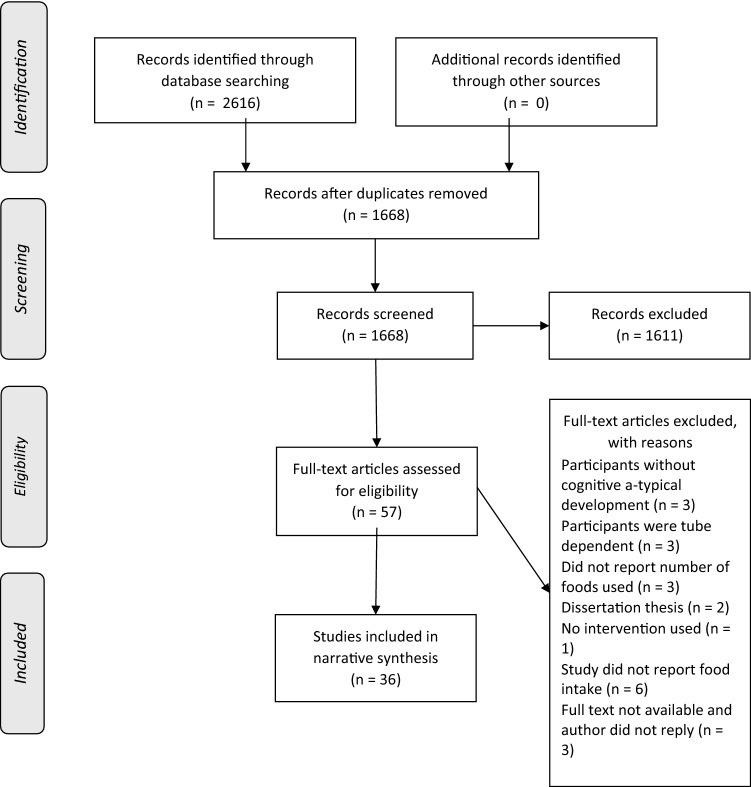
PRISMA flow chart of the study selection procedure

Two thousand six hundred and sixteen studies were identified using the search strategy. Retrieved studies were initially searched for duplicates which left a remaining 1668 papers to be screened. Titles and abstracts were screened in full by one reviewer and 5% by two separate reviewers (2.5% each; *n* = 84) who were blinded to the first reviewers’ decisions. Of these double-screened papers, five were identified for further screening and agreement was 100% between reviewers. Fifty-seven papers in total were identified for full text screening. At this stage, all papers eligible were screened in full by one reviewer and 50% each by two independent reviewers. The decision of each reviewer was blinded from other reviewers, but the studies being screened were not blinded to author or journal information. Disagreements between reviewers were resolved by consensus and agreement rate was 83%. In total, 21 papers were removed due to not meeting the eligibility criteria, leaving 36 studies to be included in the review (PRISMA flow diagram; Fig. 1).

### Data Collection Process

A data extraction sheet (Cochrane Public Health Group Data Extraction and Assessment Template 2011) was modified to suit the needs of the current review. This was tested on five papers at the full text screening stage and refined accordingly. One reviewer extracted data for 100% of the studies and this was checked by two other reviewers. Disagreements were resolved by discussion between two reviewers and if necessary, it was planned that the third reviewer would have final say on resolving disputes.

### Data Items

Extracted information from each included study was based on the relevant PICOS criteria. This included key characteristics of the study (country of study and setting of the study), study design, participants involved (age, gender and DD diagnoses), characteristics of the intervention (strategies used, focus of the intervention and duration) and study outcomes (number of foods eaten, percentage of bites accepted, measured intake).

### Risk of Bias

Risk of bias for individual studies was assessed using the Single-Case Design-Risk of Bias tool (SCD-RoB) developed by Reichow et al. (2018). This tool was chosen as it allowed the reviewers to evaluate the validity of the findings in single-case research designs and compare the study quality with other research designs. The tool assesses each study for selection bias (sequence generation and participant selection), performance bias (blinding of participants and personnel and procedural fidelity) and detection bias (blinding of outcome assessors, selective outcome reporting, dependent variable reliability and data sampling) (Reichow et al. 2018). For each of these criteria, the risk is reported as low, high or unclear.

### Summary Measures

Primary outcome measures included number of foods eaten or change in the variety of foods eaten (especially number of novel foods eaten reported from different food groups), actual food intake (measured in either grams or bites eaten), percentage of food accepted from food offered and self-reported food intake (food diaries).

### Synthesis of Results

The outcomes of included studies were synthesised to identify key characteristics of interventions used (study design, duration, setting and techniques used in intervention) and the effectiveness of these interventions to increase healthy eating in DD populations. The studies were grouped based on the intervention used and outcome measures reported. It was decided that quantitative analyses, in the form of a meta-analysis, was not appropriate due to the types of study design that were included in the review and the large variance in the interventions implemented in the studies.

## Results

### Study Selection

The PRISMA flow diagram (Fig. 1) provides an overview of study selection with reasons for exclusion. Searches of Medline, Embase, Psychinfo, CINAHL and Web of Science returned 2616 results and after duplicates and any papers before the year 2000 were removed, 1668 references remained. Of these, 1611 were not relevant to the current review and 57 were full text screened. A further 21 papers were excluded, including three that did not have participants with DD and a further three studies with tube dependent participants. Three studies did not report the number of foods used, six did not report food intake and one study did not implement an intervention. A further two studies were excluded for being unpublished dissertations or theses and the full text for three papers were not accessible online and the authors were not contactable. A total of 36 studies were subsequently identified to be included in the review.

### Study Characteristics

Characteristics of included studies are summarised in Table 2.

**Table 2:** Study characteristics and healthy eating outcomes for included intervention studies

Study ID (First author, year published, country)	Study characteristics	Participants (N, age, gender and diagnoses)	Setting	Interventions used	Duration of intervention	Measures used						Outcomes	Follow-up
						% Bite acceptance	Number of foods eaten	Number of bites	Behavioural demands	Weighed intake	Self-report		
Ahearn (2003), USA	Case study, multiple baseline	N: 1 Age: 14 Gender: M Diagnoses: autism (profound)	Clinical	Adding condiments (simultaneous presentation) to 3 disliked vegetables)	38 Sessions	✓						Increased from 0 to 20% (baseline) to 100% (intervention)^a^	2-Week diet history 1-year after intervention
Allison et al. (2012), USA	Case study, multiple baseline	N: 1 Age: 3 Gender: M Diagnoses: Autism	Clinical	DRA + EE versus NCR + EE.	38 Sessions	✓						Increased 0% (baseline) to 100% (intervention)^a^	N
Barahona et al. (2013), USA	Case study, two baselines and two interventions	N: 1 Age: 18 Gender: F Diagnoses: Autistic disorder and moderate ID	SEN School	Demand fading, verbal positive reinforcement, sticker reinforcement	49 Sessions		✓					Increased acceptance of 5 novel food items. Regular home packed meals fully replaced with novel foods at end of intervention^a^	N
Binnendyk and Lucyshyn (2009), USA	Case study with family.	N: 1 Age: 6 Gender: M Diagnoses: autism	Clinical/home	PBS with family and intervention including: stimulus fading, shaping procedures, NCR and EE	22 therapist sessions and 12 probe sessions with parents	✓	✓					Acceptance with therapist increased from 0% (baseline) to 100% (post-intervention) with 5 foods. % of food consumption with family increased from 0% (baseline) to average 64% (post-intervention)^a^	5 follow up sessions up to 2-years post intervention
Brown (2002), UK	Case study, multiple baseline	N: 1 Age: 7 Gender: M Diagnoses: moderate Learning disabilities	Clinical	Using new foods that are similar to those previously accepted, portion fading, NCR and verbal reinforcement	Daily intervention lasting 3-months		✓					Amount of a new foods eaten increased from 0 chips eaten to 10 chips at 3 month FU, 0 to 1 bread roll at FU and 1 to 4 different flavoured yoghurts eaten at FU^a^	3-Months
Cassey et al. (2016), USA	Case series, baseline, modified baseline and intervention	N: 4 Ages: 14, 15, 16 and 19 Gender: All M Diagnoses: PDD; ASD and ID; ASD and ADHD; ASD (respectively)	After school programme	Visual and verbal positive reinforcement using a game	15 sessions			✓				Number of bites of FV eaten increased. Baseline *M* = 0 bites, Modified Baseline *M* = 6.2 bites (no SD reported), range = 0-15.6 bites Intervention *M* = 13.8 (range = 8–31.3 bites)^a^	N
Cosbey and Muldoon (2017), USA	Case series, baseline and intervention	N: 3 Ages: 6, 7 and 8 Gender: All M Diagnoses: All ASD	Home	Environmental intervention tasks with the family	Not reported	✓	✓					Mean % food acceptance increased. Food frequency Average = + 14 foods. + 6, + 16 and + 20 new foods eaten by each participant^a^	FU maintenance probes until 6-months
Ewry and Fryling (2016), USA	Case study, multiple baseline, intervention and parent implementation	N: 1 Age: 15 Gender: M Diagnoses: ASD	Home	High probability sequence to 2 non-preferred foods	19 Sessions	✓						Increased % acceptance of low-p foods. Baseline *M* = 0%, intervention *M* = 96%. Return to baseline *M* = 16%, return to intervention *M* = 94%. Parental implementation *M* = 96%.^a^	Two sessions at 7-months
Fernand et al. (2016), USA	Case series	N: 2 Ages: 6 and 7 Gender: F and M (respectively) Diagnoses: both ASD	Not reported	Choice of 2 of 4 non-preferred foods presented + NRS (if did not self-feed). versus NRS alone without a choice of foods	47–51 sessions	✓		✓				Choice intervention increased the number and frequency of accepted non-preferred bites consumed for one participant. This did not work for the other participant and frequency only increased when NRS was used^a^	N
Fu et al. (2015), USA	Case series, Non-concurrent multiple baseline	N: 2 Ages: 9 and 10 Gender: Both M Diagnoses: both autism	Clinical	Modelling, DRA and NRS	33 Sessions	✓						Participant 1: modelling + DRA increased percentage of bites consumed from 0% (modelling alone) to around 70%. Modelling + DRA + NRS increased this to 100%. participant 2: Modelling + DR made no difference from baseline. Modelling + DR + NRS increased intake to 100%^a^	Two sessions at 8 and 4-weeks (each participant)
Hodges et al. (2017), USA	Case series, Baseline, intervention and maintenance phase	N: 2 Ages: 7 and 8 Gender: M and F (respectively) Diagnoses: ASD; and ASD, epilepsy, ADHD, ID (respectively)	Clinical	Single food reinforcement contingency, DRA.	92–113 trials		✓		✓			Intervention gradually increased level of acceptance from total refusal at baseline through touching food to lips, putting food in the mouth and eventually swallowing the food. Number of new foods eaten increased from 0 to 4 for both participants^a^	N
Hubbard et al. (2015), USA	Quasi-experimental, pre–post design	N: 43 Age: 11–22 years Gender: 51% female Diagnoses: All ID or developmental disorder	Residential School for Intellectual Disabilities	Environment change to school lunchroom informed by behavioural economics	3-months					✓		Mean gram weight of foods consumed did not change over the study	N
Kadey et al. (2013), USA	Case series, Multiple baseline and interventions	N: 2 Ages: 3 and 9 Gender: M and F (respectively) Diagnoses: ASD; ASD and severe-profound ID (respectively)	Clinical	NRS + NCR and Physical guidance using Nuk brush to facilitate child opening mouth. Graded fading, verbal prompts, fade prompts, hand over hand guidance and Nuk prompts were also used	Various session lengths depending on participant and intervention	✓						Participant 1 0% acceptance at baseline, increased following NRS and NRS + NCR phases to 80-100% using NRS + NCR + physical guidance. Participant 2 increased acceptance for a rejected food after non-removal, fading and Nuk procedure intervention, as Nuk procedure alone did not increase acceptance^a^	107-days after intervention for one participant
Kim et al. (2018), South Korea	Pre-post experimental design. Intervention with control group	N: 27 (13 intervention group versus 12 control group) Age: 2–5 years Gender: 23 M and 3 F (11 M in intervention and 2 F) Diagnoses: intervention group all diagnosed ASD	Laboratory	Visual + tactile guided vegetable exposure through play	One activity a day, for 4 days a week (5–10 min). Total 6-months			✓				Increased from Pre intervention (*M* = 5.73, *SD* = 13.55) to Post (*M* = 19.46, *SD* = 23.84). The control group did not differ, Pre (*M* = 1.75, *SD* = 3.82) Post (*M* = .46, *SD* = 1.10)^a^	N
Koegel et al. (2012), USA	Case series, Baseline, Intervention and follow-up	N: 3 Age: 6-8 years Gender: all M Diagnoses: all autism	Not reported	Individualized reinforcers, high probability sequence, and NCR	Until 15 new foods accepted or 22-weeks		✓					Number of new foods accepted increased from 0 (baseline) to 9, 8 and 5 (for each participant) after intervention. At generalization, 15, 16 and 6 new foods accepted^a^	FU after intervention to measure generalisation
Levin and Carr (2001), USA	Case series, baseline and multiple interventions	N: 4 Ages: 5, 6, 6 and 7 Gender: 3 M and 1 F Diagnoses: all autistic disorder	Classroom	Access to preferred food pre-intervention and positive reinforcement	30-45 sessions (depending on participant)					✓		Only 3 participants took part in intervention phases. Only the no access to preferred food prior to intervention and positive reinforcement condition resulted in intake of non-preferred food^a^	N
Marshall (2015a, b), Australia	Parallel-group randomized clinical trial	N: 78 (68 in intervention) Age: 2–6 years Gender: 50 M and 18 F Diagnoses: all ASD	Clinical	Operant conditioning (faded verbal and visual prompts with social reinforcement) versus systematic desensitisation (modelling and play based, with social reinforcement)	Ten sessions, either weekly (10-weeks) or intensive (10 sessions in 1 week)		✓					Total food count, FV count, carbohydrate count and protein counts increased in both OC and SysD intervention groups. There were no significant differences between weekly and intensive intervention models^a^	3-months
Miyajima et al. (2017), Japan	Before and after self-control study	N: 23 Age: 3–6 years Gender: 18 M and 5 F Diagnoses: 19 ASD, 4 other developmental disorders	Not reported	Psychoeducation during parental training	Two 40-min sessions and two discussions for the parents of children with ASD		✓					The number of food items consumed (out of 47 foods) increased from 20.52 to 25.17 items (p = .004) after intervention. The number of unaccepted food items decreased from 14.52 to 11.79 items after intervention (p < .001^a^	N
Muldoon and Cosbey (2018), USA	Case series. Pre-post design	N: 3 Ages: 3, 4 and 5 Gender: all M Diagnoses: all ASD	Clinical	Mealtime plans, parental training and behavioural strategies based on the EAT-UP model	6 months		✓				✓	Food frequency questionnaire (results not reported) and 24 h Recall reported that all participants increased the variety of foods eaten in a 24-h period and diets included previously non-preferred foods^a^	The study is a FU
Najdowski et al. (2003), USA	Case study, multiple baseline	N: 1 Age: 5 Gender: M Diagnoses: ASD	Home and Restaurant	Demand fading, DRA and DRA + EE + demand fading	79 meals		✓	✓				During baseline and DRA, participant never accepted or swallowed non-preferred foods. During DRA + EE + demand fading, participant accepted (but expelled) one bite of non-preferred food and began swallowing bites during the fifth meal. At home, the participant eventually swallowed 62 bites of five different non-preferred foods (Baseline = 0–5 bites)^a^	2, 4, 6 and 12 weeks after intervention
Najdowski et al. (2010), USA	Case series. Multiple baseline, intervention and generalisation	N: 3 (only 2 with a-typical development) Ages: 2 and 4 Gender: F and M (respectively) Diagnoses: Both Autism	Home	Home-based Parental training. Mothers implemented DRA + EE at baseline and DRA + NRS + Demand Fading at intervention	Up to 49 sessions	✓						All participants increased percentage of swallowed non-preferred target foods after intervention phase^a^	2, 4, 6 and 12 weeks after intervention
Patel et al. (2007), USA	Case study, multiple baseline	N: 1 Age: 4 Gender: M Diagnoses: PDD	Clinical	High-probability instructional sequence	28 sessions including 3-month follow-up				✓			Compliance to low-probability (low-p) requests was zero when low-p (spoon with food) instructions were presented in isolation. This increased to 100% when the high-p sequence (empty spoon) preceded low-p instructions^a^	3-months
Paul et al. (2007), USA	Case series, baseline and intervention	N: 2 Ages: 3 and 5 Gender: M and F (respectively) Diagnoses: both autism	Clinical	Repeated taste exposure, EE and portion fading	13–15 days and 3-month FU		✓				✓	Number of foods eaten increased from two foods at baseline to 65 foods after intervention and 53 foods (reported by parents) at FU. The other participant increased from 0 foods at baseline to 49 foods after intervention and 47 foods (reported by parents) at FU^a^	3-months
Penrod et al. (2010), USA	Case series. baseline and multi-element design	N: 3 Ages: 4, 4 and 5 Gender: all M Diagnoses: sensory, visual-motor and oral-motor delays; ASD; and ASD (respectively)	Clinical and Home	High-probablility sequence, simultaneous presentation of high and low probability foods and prompts NRS and bite fading procedures were added to the intervention	Not reported	✓						All participants showed an increased percentage of bites consumed for most previously Non-preferred foods^a^	N
Penrod et al. (2010), USA	Case series. Multiple-baseline, and phased intervention	N: 3 Ages: 3, 4 and 4 Gender: All M Diagnoses: autism; autism; and PDD (respectively)	Home	Parent delivered intervention: Phase 1: DRA + Escape + bite fading. Phase 2: DRA + Escape + bite fading + reinforcer manipulation. Phase 3: DRA + bite fading + reinforcer manipulation + EE	Up to 129 sessions			✓				Participants 1 and 2 never accepted or swallowed any food until phase 3. Participant 3 accepted a small number of bites at phase 1 and consistently accepted and swallowed non-preferred foods at phase 2^a^	12-weeks
Pizzo et al. (2012), USA	Case study, baseline and intervention	N: 1 Age: 16 Gender: M Diagnoses: ASD	Clinical	Sequential presentation (plate A–plate B)	Up to 55 meals	✓	✓					Participant met mastery criterion for 14 new foods; six starches, two dairy, two fruit, one vegetable, and three proteins. Percentage of successful bites increased from 0% (baseline) to an average of 74% during intervention^a^	N
Seiverling et al. (2018), USA	Case series, alternating treatments design.	N: 2 Ages: 5 and 6 Gender: both M Diagnoses: both ASD	Clinical	Behavioural feeding interventions with and without Sensory Integration Therapy condition NRS was used if non-compliant	24-30 daily sessions	✓						Percentage of bites was less than 40% (baseline) and increased to above 90% during sensory integration therapy and control conditions. Both participants increased total amount consumed across both treatment conditions^a^	2-months (only for one participant)
Seiverling et al. (2012a), USA	Case study, baseline and intervention	N: 1 Age: 3 Gender: M Diagnoses: ASD	Clinical	NCR (plate A–plate B) and DRA, EE and demand fading (size of bites)	Five days from approximately 8:30 AM to 4:30 PM as part of an intensive day treatment program. 35 sessions	✓	✓					Percentage of accepted bites increased from 0% (baseline) to 100% when using EE. Number of foods eaten increased from 17 to 39 (+ 22 foods)^a^	1 and 3-months
Seiverling et al. (2012b), USA	Case series, multiple baseline	N: 3 Ages: 4, 5 and 8 Gender: all M Diagnoses: all ASD	Home	Behavioural skills parent training and treatment package including taste exposure, EE and fading	11 treatment days and 3–4 weeks follow-up	✓						During baseline taste sessions, all children consistently refused bites. During post training, each child showed an increase in bites accepted^a^	Weekly FU for 3-weeks
Sharp et al. (2011), USA	Retrospective chart review	N: 13 Age: 2–8 years Gender: 11 M and 2 F Diagnoses: All ASD	Clinical	EE, NCR, DRA, and stimulus fading procedures	8-weeks, for an average total of 39 treatment days (range 29–46 days)	✓						At baseline, low rates of acceptance (*M* = 7%; range 0–48%) and swallowing (*M* = 7%; range 0–65%) were observed. There was a significant main effect for mouth cleans after treatment, *F*(1,15) = 114, *p* < 0.001, partial eta squared = 0.92. The increase in intake was seen in fruits, vegetables, proteins and six starches. Wilcoxon signed rank (Z = − 2.972; p < 0.003)^a^	N
Silbaugh and Falcomata (2017), USA	Case study, reversal design	N: 1 Age: 4 Gender: M Diagnoses: ASD	Home	Lag schedule of positive reinforcement and NCR	30 sessions	✓	✓					A decreasing trend in consumption (*M* = 52.5%; range 20–90%) and variety (*M* = 2.5; range 2–4) was observed across sessions during lag 0. Lag 1 increased consumption relative to lag 0 but with variability (*M* = 53.3%; range 20–90%); variety of foods consumed also increased (*M* = 3.06; range 2–4)^a^	N
Silbaugh et al. (2017), USA	Case study, baseline and multiple graded interventions	N: 1 Age: 3 Gender: F Diagnoses: ASD	Home	Lag schedule of positive reinforcement and Response blocking of invariant consumption, least-to-most prompting and EE	36 sessions	✓	✓					No independent consumption was observed during baseline or Lag1/toys conditions. Lag 1/toys/least-to-most prompting/response blocking increased percentage consumption (*M *= 43%). Lag 1/Response blocking increased variety of foods consumed (*M *= 2.3). During Lag 2/RB, the variety of food consumed increased from a mean of 2 foods to a mean of 3.3 foods^a^	N
Tanner and Andreone (2015), Canada	Case study, baseline and intervention	N: 1 Age: 3 Gender: M Diagnoses: ASD	Clinical	Graduated Exposure and DRA.	6-months/100 sessions		✓					Increased food acceptance from four foods to over 50 foods, with 27 of those foods generalizing to additional settings and people^a^	N
VanDalen and Penrod (2010), USA	Case series, multiple baseline combined with multi-element intervention	N: 2 Ages: 4 and 5 Gender: both M Diagnoses: both ASD	Clinical	Simultaneous presentation of a non-preferred food with a high-preference food and sequential presentation. NRS with a schedule of reinforcement was also used	Between 55 and 64 sessions	✓						At baseline neither participant consumed non-preferred foods. During simultaneous and sequential presentation, minimal bites were accepted or consumed. When NRS was adopted, 80–100% of bites were consumed^a^	1-year
Wallen et al. (2013), Sweden	Cross-sectional, intervention versus control	N: 89 (27 in intervention group) Age: 16–21 years Gender: Intervention group = 15 F and 12 M, Control group = 29 F and 33 M Diagnoses: all moderate/mild ID, 12 with Down’s syndrome and 3–6 with ASD (ASD rates not well reported)	School	School environmental intervention using modified plates and psychoeducation	Minimum 6-months		✓			✓		No significant differences observed between groups based on food choice, plate waste or intake, including FV intake	N
Wood et al. (2009), USA	Case study, multiple probe design	N: 1 Age: 5 Gender: M Diagnoses: Autistic disorder	Home	Combined task direction, NCR, physical prompts (hand over hand), and demand fading	39 sessions	✓						At baseline, only already accepted foods were eaten. During intervention, bites of non-preferred foods increased to 100%, until bites presented increased from three to six per session. Foods never eaten at baseline increased to 50% bites consumed^a^	N

*ASD* autism spectrum disorder, *ID* intellectual disorder, *PDD* pervasive developmental disorder, *DRA* differential reinforcement of alternative behaviour, *EE* Escape extinction, *NCR* non-contingent reinforcement, *NRS* non-removal of the spoon, *PBS* positive behavioural support, *OC* operant conditioning, *SysD* systematic desensitisation, *FU* follow up, *FV* fruit and vegetables

^a^Study was effective

#### Methods

Thirty of the 36 included studies were case studies or case series. Three used pre–post intervention designs, one study used a cross sectional intervention versus control design, one examined a retrospective chart review and one study was a parallel-group randomized clinical trial. 30 studies were conducted in the USA, with one study each conducted in the UK, Canada, Sweden, Australia, South Korea and Japan.

#### Participants

A total of 317 participants took part across the 36 included studies, with the majority being male (*n* = 217). As age was not conditional, the studies included participants with an age range of 2 to 22 years. Most studies included children between 2 and 8 years, although a few studies included age ranges of 16–22 years. Participants were also mostly diagnosed with ASD, with a few studies including participants with a diagnosis of ID, pervasive developmental delay, global and specific developmental delays, Down’s syndrome and ADHD.

#### Intervention

Thirty-two studies were evaluated on pre-intervention and post-intervention measures, and four studies included either a control group or a parallel intervention group. Studies rarely used one intervention alone and most interventions were implemented as a package with multiple strategies used. Techniques used in interventions are described in Table 3.

**Table 3:** A description of the intervention techniques used and how many studies in the current review used the technique

Method/intervention	Description	Number of studies
Based on operant conditioning		
Escape extinction (EE)	This technique describes various procedures that prevent escape from the feeding situation (including non-removal of the spoon: NRS and physical guidance; Piazza et al. 2003)	18
Non-removal of the spoon (NRS)	A type of EE that holds a spoon close to the mouth until the food is accepted	7
Physical guidance	Guiding the mouth open or applying small pressure to the jaw to assist with accepting food into the mouth	2
Differential reinforcement of alternative behaviour (DRA)	Positive reinforcement of ‘target’ or ‘good’ behaviours (e.g. reinforced with food, toys, stickers and verbal praise) on a variable schedule (Piazza et al. 1996)	14
Non-contingent reinforcement (NCR)	Reinforcement that is not dependent on completing a ‘target’ behaviour (e.g. swallowing a non-preferred food item)	9
Lag schedules	A schedule of reinforcement in which a single response, or a sequence of responses, is reinforced if it varies from previous responses or sequences of responses (Page and Neuringer 1985)	2
Based on exposure		
Systematic desensitisation (SysD)	A method designed to reduce avoidance behaviour towards an adverse stimulus by gradually increasing exposure to it (Davison 1968)	15
Stimulus/texture, Portion and Demand fading	Three methods of SysD Stimulus/texture fading: Gradually changing the texture of a food (e.g. runny mashed potato can be made increasingly thicker) Portion fading: Gradually increasing a portion of a new food (e.g. from a pea size to a recommended serving) Demand fading: Gradually increasing behaviours that are required by the participant (e.g. increasing the demand from one bite to three bites)	3, 2 and 7 respectively
Simultaneous presentation	A type of flavour–flavour conditioning that pairs a non-preferred food with a preferred food or liked condiment	3
Using new foods similar to those previously accepted	Using similar foods to those already accepted (e.g. matching by food group, brand, colour, texture etc.)	1
Modelling	Watching others eat the non-preferred food (e.g. parents, siblings, friends)	2
High probability sequences	This requires asking the participant to complete a high-probability task (e.g. put spoonful of preferred food in the mouth) before asking them to perform a low-probability task (e.g. put a spoonful of non-preferred food in the mouth) (Ewry and Fryling 2016)	4
Choice of foods	Allowing the person a choice between different non-preferred foods (Fernand et al. 2016)	1
Access to preferred food	The preferred food is offered before the non-preferred food is presented	4
Family and environmental methods		
Psychoeducation	Psychoeducation involves providing education and information to family members about selective eating in the DD population	2
Parental training	Most of the techniques described are implemented by clinicians or researchers. Parental training is designed so that parents can implement some strategies themselves	6
Mealtime plans	Mealtime plans are implemented by the family and focus on areas such as communication, food, social and physical environment during mealtimes (Muldoon and Cosbey 2018)	1
Positive behavioural support (PBS)	A multi-component intervention that aims to support an individual with DD. This includes a functional assessment of possible relationships between environment and behaviour, which can inform support for eating using appropriate methods (methods in this table) for the individual (Binnendyk and Lucyshyn 2009)	2
Environmental interventions	Environmental interventions used by studies in the current review included changing the layout and placement of healthy foods in lunchrooms, using special plates that show how much of the plate should be filled with portions from each food type and using team games to encourage snack FV intake	4

#### Setting

Nineteen studies were carried out in clinical or laboratory settings, nine were set in the home and a further five studies took place in school settings (see Table 2). The remaining three studies did not report where interventions took place.

#### Outcomes

All studies collected and reported outcomes related to food intake and 19 studies reported these values for baseline, post-intervention and at follow-up time points. Measures of food intake included total number of foods eaten, number of pieces of foods eaten (pieces were sometimes defined as 1.5 cm^3^ of food; Cassey et al. 2016), percentage of accepted (consumed) bites during a meal, reported intake from food diaries, measured weight of food consumed (grams) and behavioural demands, such as picking up food, touching the lips and eventually swallowing the food.

### Risk of Bias Within Studies

Table 4 outlines the risk of bias judgements of the reviewer for the 36 included studies. For the current review, some of these criteria were not applicable to individual study designs. Using the SCD-RoB tool, case studies included in the review were generally rated as low-risk of bias, whereas other study designs were rated as having a higher risk of bias.

**Table 4:**
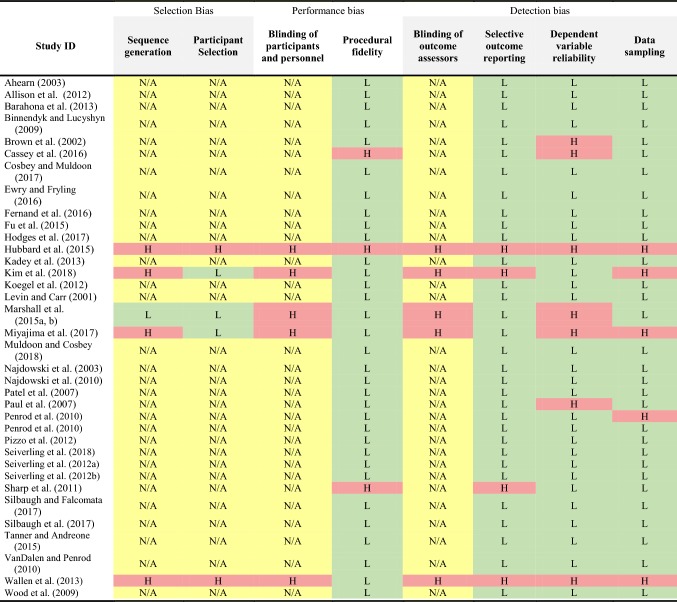
Assessment of study quality using the SCD-RoB tool (Color table online)

### Synthesis of Results

A narrative synthesis of included studies was carried out. This details the common themes that occur and clusters studies based on the interventions used and their outcome measures.

### Theory Driven Interventions

Interestingly, 34 of the 36 studies retrieved reported having positive or effective results. All studies except three were grounded in learning theory to shape eating behaviour. Two studies not based on learning theory used environmental changes grounded in behavioural economic strategies (Hubbard et al. 2015; Wallen et al. 2013), neither of which were effective. The third delivered psychoeducation to parents (Miyajima et al. 2017). This study was effective at increasing number of foods eaten (a mean increase of five foods).

Learning theory, based on the principles of classical and operant conditioning, was the most common theme throughout the interventions used. In total, 21 studies included a behaviour reinforcement component that was central to the intervention, with many more studies involving positive reinforcement as usual practice after a target behaviour was achieved. Of these, 14 studies used DRA (differential reinforcement of alternative behaviour) and nine used NCR (non-contingent reinforcement) procedures to reinforce target behaviours during eating. Procedures included a range of personalised reinforcers, including access to preferred toys (Binnendyk and Lucyshyn 2009) and preferred foods (Seiverling et al. 2012a), to reward the target behaviour (eating a novel or non-preferred food). Both techniques were effective when used as part of an intervention and some interventions used DRA and NCR techniques together (e.g. Seiverling et al. 2012a).

Eighteen studies used EE (escape extinction) techniques to prevent participants from avoiding the target food and seven of these EE studies employed NRS (non-removal of the spoon). EE procedures were reported to be effective for increasing acceptance of bites in all studies that used the technique *(*Allison et al. 2012). In contrast to this method, exposure, systematic desensitisation and various fading techniques (texture, portion, and demand) were used in 15 studies as a means of gradually exposing the participant to more of the food stimulus, or different textures of the same food stimulus. These techniques were grounded in learning theory in the same way as EE, however they were based on gradual introduction to the novel stimulus rather than a flooding experience of the food stimulus. Increasing exposure was also effective at increasing intake of new and disliked foods, although exposure on its own may be less effective than EE to increase eating of new foods in selective eaters (Kim et al. 2018).

Four studies employed scaffolding techniques to prompt appropriate responses from participants. This included physical (e.g. hand over hand) and verbal prompts, encouraging the participant to carry out the target behaviour themselves by showing them what to do (Wood et al. 2009). Similarly, two studies employed social learning theory in the form of modelling eating as part of an intervention package, which was reported as having limited effectiveness (Fu et al. 2015).

### Single Versus Multiple Component Interventions

The majority of studies combined components during interventions, with only two studies purely using single-component interventions to increase eating outcomes. Patel et al. (2007) and Ewry and Fryling (2016) both reported that high-probability instructional sequences increased behavioural outcomes and percentage of bites accepted when compared to low-probability instructional sequences. All other 34 studies used multiple-components in their interventions, although five studies phased their approach to implementing interventions and therefore illustrate that some interventions were not effective when presented alone. Fu et al. (2015) found that modelling did not increase intake from 0%, but when DRA was implemented together with modelling, intake increased to 70% and finally 100% when NRS was also introduced. Similarly, Kadey et al. (2013) reported that NRS and NCR procedures together did not increase acceptance of novel foods for one participant, but these outcomes increased when physical guidance was added.

EE and NRS techniques were generally effective for increasing consumption of bites offered and these techniques were usually added when other strategies did not work. For example, Najdowski et al. (2003) illustrated that DRA and demand fading did not have effective outcomes until EE was added to the intervention. Similarly, Penrod et al. (2010) showed that adding EE to the intervention resulted in swallowing new foods for two participants. For the third participant, DRA, escape and bite fading already resulted in some acceptance, so when reinforcer manipulation was added, intake increased further and EE was not needed. These reports suggest the need for a hierarchy of interventions to apply when particular techniques do not achieve a meaningful increase in acceptance in selective eaters. It appears that when reinforcement or systematic desensitisation techniques are not effective, EE and NRS can be effective at increasing intake especially when the participant is extremely resistant to other methods. However, if this does not work, physical guidance may lead to positive outcomes such as accepting a non-preferred food, as illustrated by Kadey et al. (2013).

### Micro Versus Macro Outcomes

Reported outcomes for each study also varied, with 24 studies reporting percentage of food accepted or number of bites consumed and 17 studies reporting total number of foods eaten and the number of new or disliked foods that were eaten before and after intervention. There were also differences within outcome measures reported. Total number of foods eaten was sometimes reported as number of food items and at others as number of pieces of that food item (e.g. how many chips were eaten; Brown et al. 2002). The number of new foods eaten ranged from + 2 to + 63 foods, although the majority of interventions reported between +5 and +15 foods. This is in contrast to the percentage of bites eaten from 0 to 100% of a single new food.

Interestingly, many different interventions, including graduated exposure, DRA, fading techniques and EE, were all successful for increasing the number of new foods eaten (Marshall et al. 2015a; Paul et al. 2007; Tanner and Andreone 2015), whereas for increasing intake, DRA, EE, NCR, high probability sequences and physical prompts were all successful at increasing percentage of bites accepted (Allison et al. 2012; Pizzo et al. 2012; VanDalen and Penrod 2010). This illustrates that although different outcomes were measured, similar techniques were used whether the target was on a macro (whole new foods eaten) or micro scale (bites of a new food presented). Exposure techniques were more commonly used when attempting to increase the number of new foods eaten (Barahona et al. 2013; Brown et al. 2002) and EE techniques were employed in most effective interventions increasing acceptance of bites (Fernand et al. 2016; Najdowski et al. 2010). Nevertheless, both techniques were successful for both outcomes.

### Duration of Intervention

Duration of interventions were reported either in time or number of sessions, although some studies did not report this clearly or did not state the intensity of delivered sessions (Cosbey and Muldoon 2017). Studies lasted from 15 sessions (Cassey et al. 2016) to 129 sessions (Penrod et al. 2010), or from 5-days (Seiverling et al. 2012) to a minimum of 6-months (Wallen et al. 2013). Other studies set goals before termination of the intervention, such as until 15 new foods were accepted or until 22-weeks had elapsed (Koegel et al. 2012).

Furthermore, only 19/36 studies had a follow-up. This ranged from weekly at 2, 4, 6 and 12-weeks (Najdowski et al. 2003, 2010) to the longest follow-ups at 1-year (VanDalen and Penrod 2010) and 2-years (Binnendyk and Lucyshyn 2009). However, 17 studies did not have (or did not report) a follow-up time-point to evaluate the long-term effects of the studies. Of the studies that did follow-up, there was some loss of performance, but outcomes were generally better than baseline. Binnendyk and Lucyshyn (2009) reported 100% acceptance of bites offered at post intervention, but this reduced to 64% up to 2-years follow up. Outcomes from Paul et al. (2007) also reduced from 65 new foods eaten post-intervention to 53 at 3-month follow-up. However, some studies maintained benefits of the intervention at follow-up and in one study, the number of foods eaten continued to increase at follow-up compared to post intervention (Koegel et al. 2012).

### Setting

Of the 19 studies carried out in clinical or laboratory settings, all reported positive effects. These interventions tended to include DRA, EE and NCR procedures and were generally longer in session duration, whereas the nine studies carried out in home settings (although still by a clinician) tended to include prompts, fading techniques and positive reinforcement. After clinician implemented interventions, a few studies used parental implementation of the same interventions which appears to be effective. Ewry and Fryling (2016) report that percentage of bites accepted (of non-preferred foods) maintained at 96% during parental implementation. Similarly, studies where only parents and not clinicians implemented interventions (after training) were also reported to be effective (Penrod et al. 2010; Seiverling et al. 2012b).

The remaining five studies took place in school settings. Two of these studies implemented environmental changes, such as portion-size modified plates, (Hubbard et al. 2015; Wallen et al. 2013) that were not effective. The other three studies implemented access to preferred foods and positive reinforcement (Levin and Carr 2001), demand fading and positive reinforcement (Barahona et al. 2013) and positive reinforcement using a game (Cassey et al. 2016), which were all reported as effective.

## Discussion

This review was conducted to identify types of interventions used for increasing dietary variety and acceptance of fruits/vegetables in DD populations and to determine their effectiveness. It was found that a range of techniques have been used to increase dietary variety, which can be categorised into three groups based on operant conditioning, systematic desensitisation and environmental/family based interventions. Techniques from all of these groups have been reported to be effective (although environmental interventions were only effective when combined with family interventions) for increasing healthy eating on an individual, case-by-case basis, by increasing the number of new foods eaten, the percentage of bites accepted during a meal and the amount (weight) of new foods that have been consumed. It was found that many studies attempting to increase dietary variety applied a package of different interventions spanning the three categories, to encourage change in acceptance of food.

Generally, interventions were reported to be most effective when multiple components were used, there was an incentive through reinforcement such as access to toys, or when systematic desensitisation or escape extinction procedures were used. Multi-component and phased interventions also suggest that for each individual, there is a hierarchy of techniques that might be effective to increase acceptance and dietary variety. Although EE techniques have been consistently reported as most effective, exposure and reinforcement techniques should be tried before EE and physical guidance strategies due to ethical reasons and to avoid the possibility of adverse side effects of EE (Goh and Iwata 1994). This suggestion fits well within a clinical model, however it can be questioned whether multiple component interventions are more commonly implemented because individual cases are complex, or whether it is because some interventions simply do not work (for the individual or the population).

Furthermore, although effective, nearly half of the studies did not follow-up participants after the intervention, meaning that the long-term effectiveness of these interventions cannot be determined. Although in some instances follow-ups may not be possible, this is an important detail that is missing from the literature because if there is no continued benefit after the intervention stops, then it could be questioned whether these interventions would warrant being implemented. This is especially important due to the length of some studies without a reported follow-up (e.g. 113 trials; Hodges et al. 2017) as this may be deemed excessively long without examining or reporting the lasting effectiveness of the intervention. Possible confounding variables (e.g. a maturation effect) are also often not considered as a possibility for decreasing selective eating or food refusal (Bandini et al. 2017) in such long-term interventions.

Perhaps the most practically useful outcome from this review is that some environmental interventions, such as using special plates and changing the placement of foods in lunchrooms, are simply not effective in increasing diet variety in the DD population (Hubbard et al. 2015; Wallen et al. 2013). Although, the quality of these studies was poor (due to selection and detection biases) and it may be that better designed studies could have positive effects. Study design in general was highlighted as a major issue in this review for determining effectiveness of interventions. The review included only one randomized clinical trial, suggesting that number of foods eaten can be increased through ten-sessions of either operant conditioning or systematic desensitisation. However, 30 of the remaining studies were case studies or case series, which meant that the interventions delivered were highly personalised and could have included some of the more selective participants from the DD population, as they warranted clinical intervention. Consequently, the findings of these studies may not be generalizable to selective eaters drawn from the DD population unless they warrant clinical intervention. For these people, there is very little research specifically focused on eating a varied diet. It is only when the selective eating becomes a problem to physical health that interventions are delivered. This contrasts with healthy eating interventions in typically-developing people as these are generally aimed at improving public health and not predicated on clinical need (Mikkelsen et al. 2014). The current review helps to illustrate this difference as 33/34 positive studies were grounded in learning theory, involving strategies based on the use of applied behavioural analysis (ABA; Virués-Ortega 2010), a technique frequently used with people with ASD to shape preferred behaviours. Therefore findings of the current review are restricted to commenting on the individual with selective eating, rather than the population of selective eaters.

## Limitations

Overall, the evidence was not sufficiently robust to determine the effectiveness of these strategies on a population level. Interventions were personalised and used multiple-components in different combinations, meaning that their effectiveness could not be compared adequately.

A central limitation of this review is the Risk of Bias (RoB) within and between studies. The studies were mostly rated as low RoB using the SCD-RoB Tool. However, many studies did not report the intensity or duration of intervention, intervention setting and many did not have a follow-up time point. All of which would suggest a high RoB that was not detected by the tool used. This also indicates that there is a high risk of selective outcome reporting. Similarly, there is a risk of publication bias due to the studies mostly utilising single-case designs and reporting high rates of effective interventions. This is particularly problematic because we do not have data for how many other participants interventions were tried within clinics but not effective and therefore not published.

Lastly, many studies included reducing problem mealtime behaviours as a secondary outcome. It was beyond the scope of the current review to examine these outcomes, although it would have been useful to use this information as a measure of disgust to the novel food stimuli. This is because it is difficult to determine from study reports how selective each person was before the intervention or what characteristics individuals may have that made them more or less resistant to change. Additionally, it is impossible to tell whether the outcome measure (number of foods consumed or percentage of bites eaten) was chosen based on how resistant to change or selective the participant was, or whether the measure was chosen based on feasibility, convenience or parental concern. Dependent on the reason, it could be questioned why the effectiveness of the same interventions has been measured using both macro and micro measures. This is because if the participant is more selective, it could be easier to show intervention effectiveness based on percentage of bites accepted rather than whole foods eaten.

## Conclusion

Multiple component interventions based on operant conditioning, systematic desensitisation and combined environmental and family based interventions appear effective when applied to the individual. However, the effectiveness of these interventions for the population cannot be determined due to the majority of studies retrieved using a single-case design and employing different combinations of interventions in each study. Of the interventions identified, environmental changes at mealtimes appear to be less effective and less researched in this population, whereas behavioural interventions grounded by learning theory, such as systematic desensitisation, reinforcement and EE appear to be effective at increasing intake for a variety of novel foods and are therefore in greater evidence among published papers. However, it should be noted that there is a high risk of selective outcome reporting, publication bias and bias within and between studies included in this review.

## Future Recommendations

There is a lack of interventions for DD populations to improve specifically variety of foods accepted and in particular FV intake. Systematic interventions with aims, duration and outcome measures laid out before delivering the interventions (pre-registration) are needed to determine whether individual interventions are effective. The interventions identified are also mostly used with DD populations, therefore it would be useful to determine whether interventions in which have been successful in typically-developing populations are also effective for people with DD, without clinically significant nutritional deficiencies. Lastly, it is important to establish whether these interventions are effective when delivered to a group or on a one-to-one basis, as many successful interventions to increase acceptance of novel foods in typically-developing populations are implemented in schools and not by clinicians.
